# Online Fake News about Food: Self-Evaluation, Social Influence, and the Stages of Change Moderation

**DOI:** 10.3390/ijerph18062934

**Published:** 2021-03-12

**Authors:** Greta Castellini, Mariarosaria Savarese, Guendalina Graffigna

**Affiliations:** 1 Faculty of Agriculture, Food and Environmental Sciences, Università Cattolica del Sacro Cuore, via Milano 24, 26100 Cremona, Italy; mariarosaria.savarese@unicatt.it (M.S.); Guendalina.graffigna@unicatt.it (G.G.); 2 EngageMinds HUB—Consumer, Food & Health Engagement Research Center, Università Cattolica del Sacro Cuore, 20123 Milan, Italy

**Keywords:** online fake news, interpersonal influence, self-evaluation, motivation for change, food consumption

## Abstract

In the Italian context, the diffusion of online fake news about food is becoming increasingly fast-paced and widespread, making it more difficult for the public to recognize reliable information. Moreover, this phenomenon is deteriorating the relation with public institutions and industries. The purpose of this article is to provide a more advanced understanding of the individual psychological factors and the social influence that contributes to the belief in food-related online fake news and the aspects that can increase or mitigate this risk. Data were collected with a self-report questionnaire between February and March 2019. We obtained 1004 valid questionnaires filled out by a representative sample of Italian population, extracted by stratified sampling. We used structural equation modelling and the multi-group analyses to test our hypothesis. The results show that self-evaluation negatively affects the social-influence, which in turn positively affects the belief in online fake news. Moreover, this latter relationship is moderated by the readiness to change. Our results suggest that individual psychological characteristics and social influence are important in explaining the belief in online fake news in the food sector; however, a pivotal role is played by the motivation of lifestyle change. This should be considered to engage people in clear and effective communication.

## 1. Introduction

Online food and nutrition information seeking is a widespread and growing phenomenon. Collecting information on vitamins, diet, nutrition, and supplement information is the main motivation that leads people to use the Internet and social media [1]. However, the Internet is not always a reliable source for information on diets and food choices. In fact, individuals are exposed to a variety of dietary and food (mis)information and lifestyle advice that may be contradictory and deviant with respect to health standards, encouraging “unhealthy behavior” [2,3,4]. For these reasons, it appears urgent to understand the factors behind believing in online food fake news to engage people in a more aware search for information and better food choices. The scenario appears particularly critical in Italy, where the belief in online fake news is creating confusion in the population. Traditionally known as the standard-bearer of the Mediterranean diet, widely recognized as healthy, in latest years Italy is living a change in people’s food choices that risks to move them away from proper dietary standards [5]. In fact, the presence of misinformation in the food sector determines the creation of negative attitudes and opinions toward certain types of food (as happened for example for dairy or gluten consumption), which impact on purchase intentions and consequently can determine a permanent change in daily food habits. In order to limit this dangerous phenomenon, scholars have tried to understand what factors could contribute to the spread and belief in online fake news, and, consequently, to change people’s food choices [6,7]. In particular, many scholars have studied the role of individual psychological and social factors in determining the believing in fake news [8]. However, these variables seem not enough to explain a change in behavior due to misinformation. To propose a more complex explanation of this phenomenon, we introduce the motivation to change as a possible pivotal variable. From the literature, indeed, it is known that people in different stages of change could be more or less inclined to change their behavior toward better food choices [9], which include appropriate search for information [10]. However, up to date, the motivation to change theory has not been used to study the phenomenon of fake news. To fill these knowledge gaps, this study aims at exploring the role of motivation to change in the believing of food fake news to glimpse possible strategies to engage people in adequate food choices. 

## 2. Background and Hypothesis Development

Studying the phenomenon of online fake news in the food sector is certainly complicated because it requires the consideration of different structural, individual, and social factors, and it may be influenced by the specific life moment the individual is experiencing in relation to his own lifestyle. It has been demonstrated that individual psychological and social factors are more relevant in explaining this behavior more than structural elements, such as the time spent using social media [6]. In particular, some studies demonstrated that when people have low self-esteem and a negative perception of self-concept, they seek confirmation of their behavior in others, becoming more susceptible to interpersonal influence [11,12]. Moreover, people who are more predisposed to interpersonal influence are also more likely to believe and share the information given by others even if the information is false because this allows them to increase their social affirmation and improve their self-esteem [13]. From these premises, it is assumable that the interpersonal influence could mediate the relationships between self-evaluation and the believing in fake news. In support of this assumption, some studies affirm that it is not the level of self-esteem that directly determines the persuasive power of fake news but it is the necessity to improve self-perception that leads people to become more prone to follow the opinions and advice of others (interpersonal influence) in order to find a social consensus [14]. Moreover, as suggested by the Transtheoretical Model [15] the search for information plays a different role according to the stage of change in which the person is. In particular, this model is composed of five incremental stages of change in an individual’s behaviors and lifestyle (i.e., precontemplation, contemplation, preparation, actions, maintenance) that allow identifying different phases of lifestyle change. In particular, according to this theory, during the preparation and actions stages, individuals need to consult and interact with external information sources (such as friends, relatives, blogs, forums, etc.,) in order to be motivated to continue the change process and maintain it over time. Applying this model on lifestyle change, some scholars discovered how people modify their openness to external information and the way of seeking and using information based on specific life moment that the individual is experiencing in relation to his own lifestyle [16]. Specifically, this research discovered that people who are in a stage of changing their lifestyle (preparation and action stages) are more predisposed to being socially influenced by external sources and in particular this orientation led them to believe more in online news (even if false) than those who are not in these stages. These results pointed out how the belief in online fake news could be amplified by the motivation of change lifestyle.

From these premises, we assume that:

**Hypothesis** **1** **(H1).** *Positive self-evaluation has a negative impact on the propensity to interpersonal influence.*

**Hypothesis** **2** **(H2).** *The propensity to interpersonal influence positively influences the belief in online fake news.*

**Hypothesis** **3** **(H3).** *The study assumes that the effect of the self-evaluation on the belief in online fake news is mediated, at least partially, by the interpersonal influence.*

**Hypothesis** **4** **(H4).** *The stage of change in one’s own diet can moderate the relationship between interpersonal influence and belief in online fake news.*

**Figure 1:**
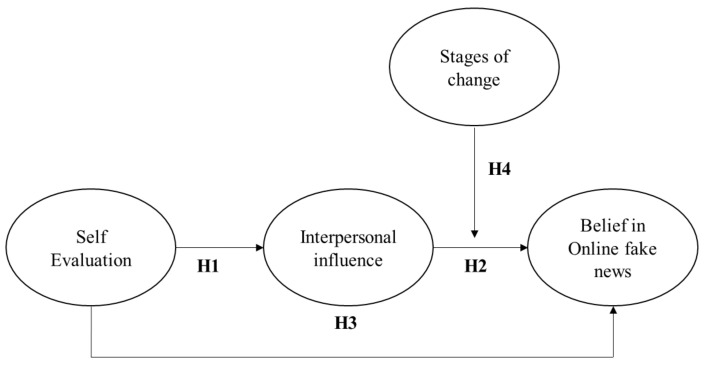
The hypothesized model.

Based on the aforementioned discussion, we can synthesize the hypothesis which inspired this study in the model depicted in Figure 1.

## 3. Materials and Methods

### 3.1. Procedure and Sample

Data were collected via an online self-administered structured questionnaire on a sample of 1004 Italians, aged 18–75 years old, representative by sex, age, profession, size of the center and geographical area, extracted by means of stratified sampling. The survey was conducted using a Computer Assisted Web Interviewing methodology in the first three weeks of March 2019. This study has been performed in accordance with the Declaration of Helsinki and has been approved by an independent ethics committee of Università Cattolica del Sacro Cuore in Milan (CERPS). All participants provided informed consent at the beginning of the questionnaire. Those who did not provide it were excluded from the database.

### 3.2. Measures

The structure of the self-administered questionnaire included both the following validated psychometric scales and ad hoc items (see Supplementary Materials):

- Core Self Evaluation Scale (CSES): we used the validation of Italian scale [17] which is composed of 12 items derived from the translation of Core Self-Evaluation Scale [18]. People who have high score on the Core Self Evaluation Scale are subjects that think positively of themselves and are confident in their own abilities. This Italian version of this scale has satisfactory reliability and validity with an α = 0.84.

All items were assessed on 5-point Likert scales ranging from 1 (“completely disagree “) to 5 (“completely agree “). An example of item is: “When I make plans, I am certain I can make them work.”

- Susceptibility to interpersonal influence: the scale is scientifically validated [19] and is composed of 12 items grouped in two factors (informational and normative interpersonal influence). Higher scores on this scale identify subjects more prone to conform to the expectations of others regarding purchase decisions, and more predisposed to learn about products and services by observing others or seeking information from others [19]. From this scale, two items per factor (normative and informative) were selected in order to reduce the quantity of questions without neglecting the measure of both factors of the scale. In particular, the chosen items are the most representative indicators of the scale as they have the higher factor loadings in the validation study. In this study, the Cronbach’s alpha coefficients for the scale were 0.83. All items are measured on 7-point Likert scales ranging from 1 (“completely disagree”) to 7 (“completely agree”). An example of item is: “If I have little experience with a product, I often ask my friends about the product.”
- Belief in Online Fake News: This behavior was assessed by an ad hoc item to explore the self-reported experience of believing in fake news that occurred in the last year. The item adopted a five-point category scale from “never” to “always.” The single-item used is: “In the last year have you believed in a piece of news about food read on the Internet or on social networks that turned out to be a fake news (Fake News)?”
- Stages of change: We based this measure on the Transtheoretical Model. In this model, five distinct motivational stages are identified [20]. This model was previously used in the Italian context on different health changes [21]. The items described here were created ad hoc on the basis of these five stages, one for each. Respondents decided to position their answer on the item that better represent their condition among the five. The item used is “How interested are you in making your lifestyle healthier than it is now?”

### 3.3. Data Analysis 

Descriptive statistics were computed for each item (asymmetry, kurtosis, mean, median, and standard deviation), and normality of distribution was checked. 

As suggested by [22] in order to check the adequacy of the measurement model a confirmatory factor analysis was run using MPLUS 8 (Muthén & Muthén: Los Angeles, CA, USA). The models were estimated using maximum likelihood estimation and evaluated using the chi-square (i.e., non-significant values associated with *p* indicate a good model) and approximate fit statistics [23]. These included: Root Mean Square Error of Approximation (RMSEA) < 0.08; Confirmatory Fit Index (CFI) ≥ 0.95; and Tucker-Lewis Index (TLI) ≥ 0.95. Moreover, the values of factor loadings, average variance extracted (AVE), and composite reliability (CR) were taken into account. In particular, factor loadings < 0.40 are weak and factor loadings > 0.60 can be considered strong [24] and the acceptable threshold value for composite reliability (CR) is above 0.70, while that for average variance extracted (AVE) is above 0.50 [25]. 

Moreover, structural equation modelling was used to analyze the relationships between the self-evaluation and interpersonal influence on the belief in online fake news. In order to control for inflated measurement errors, caused by multiple items that compose the self-evaluation latent variable, and to obtain more stable estimates and higher reliability, the use of parcels is recommended [26,27,28]. In particular, as suggested by [29] three parcels were created for representing the social-influence construct, using random assignment [30,31]. These parcels were regarded as observed variables which represent the average scores of the corresponding items. Moreover, the bootstrap technique [32] was used in order to confirm the mediation hypothesis (the indirect relationship between an independent variable and the dependent variable considering the presence of the mediator) with more statistical rigor than the Sobel test [33,34]. The Percentile bootstrapping was performed at a 95% confidence interval with 5000 bootstrap samples [35].

To explore the moderating effect of stages of change invariance tests of the measurement model and structural model were conducted, following the suggested procedures used in [36] research. Before the metric invariance test, the sample was divided in two sub-sample: in change group (n = 646) which is composed of people who have responded that they intend to change their lifestyle within the next six months, in the immediate future or who have recently changed their lifestyle; and not in change group (n = 358), considering those who responded that they have no intention of changing their lifestyle or that their lifestyles are already healthy. Then the equality between the factor loadings of both groups (measurement invariance) was performed. First, confirmatory factor analysis was employed for both groups without fixed factor loadings (configural invariance model); while another confirmatory factor analysis was conducted for both groups with fixed factor loadings (metric invariance model). Then, the two different models were compared. After that, we use the structural equation modelling in order to compare two different models (unconstrained model) and the nested model (constrained model), where in the first model all the parameters were considered free and in the second one the path coefficient between the social influence and the belief in fake news was constrained in the two groups (in change, not in change). Through this comparison, we can identify the *X*^2^ difference between the constrained model and the unconstrained one and if the *X*^2^ difference is significant, the moderating effect of stages of change exists. Furthermore, we used Welch’s ANOVA, Student’s Test-t and Contingency Tables to understand if there were socio-demographic differences between those who believe in nutritional fake news and those who intend to make their lifestyle healthier. In particular we have considered the variables age (18–39 years old; 40–55 years old; >56 years old), gender, and education level (low = no educational qualifications, elementary and Lower secondary school; medium = high school diploma; high = college or university). The age groups were created by dividing the distribution by the 33rd and 66th percentile. Moreover, to distinguish those who intended to make their lifestyle healthier from those who did not intend to change it, two groups have been created based on the question “How interested are you in making your lifestyle healthier than it is now?” People who responded that they intended to change their lifestyle within the next six months, in the immediate future, or who have recently changed their lifestyle were considered interested in making their lifestyle healthier, while those who responded that they have no intention of changing their lifestyle or that their lifestyles are already healthy were considered not interested in the nutritional change.

## 4. Results

### 4.1. Characteristics of the Sample

The sample is made up of 1004 Italian respondents of which 497 are male and 507 are female with an age between 18 and 75 years with an average age of 46 years and a standard deviation of ±15.5. The demographic profile is presented in detail in Table 1.

### 4.2. The Measurement Model

Means, standard deviations, medians, asymmetry, and kurtosis of all the scales and items ad hoc used in this study were carried out, showing that all distributions appear normal (the Kurtosis ranges from −0.69 to 0.86 and the asymmetry ranges from −0.72 to 0.62).

Moreover, confirmatory factor analysis was applied to understand whether the data confirmed the assumption that these latent variables represent two separated constructs, validating the measurement model. For this purpose, the maximum likelihood estimation method was used. 

The final measurement model included two latent constructs (self-evaluation and interpersonal influence) and seven observed variables (three parcels for the self-evaluation and four items for the interpersonal influence). Results confirm the hypothesized two-factor measurement model and all of the loadings of the observed variables on the latent variables were also significant, revealing that the latent constructs were well operationalized by their indicators (Table 2). In this model the errors of item 3 and item 4 are correlated due to the similarity of the words that compose these items (r = 0.62, *p* < 0.001). 

Table 3 shows that the estimated intercorrelations among the two latent variables (self-evaluation and interpersonal influence) were less than the square roots of the average variance extracted in each construct. This provides support for discriminant validity and thus reduces the potential influence of common method variance [37]. 

### 4.3. The Structural Model

Finally, a structural equation model was run in MPLUS 8 on the total sample (N = 1004) to assess, first, the relationships between self-evaluation and the interpersonal influence on the belief in fake news. 

The model provided a very good fit to the data: X^2^ = 50.055; df = 17; *p* < 0.001; CFI = 0.99; TLI = 0.98; RMSEA = 0.04 (LO90 = 0.03, HI90 = 0.06). In accordance with the hypothesis, interpersonal influence was negatively influenced by self-evaluation (β = −0.17, *p* < 0.001), confirming hypothesis 1, and the belief in online fake news was positively influenced by interpersonal influence (β = 0.20, *p* < 0.001), confirming hypothesis 2. 

In order to test the mediating role of interpersonal influence between the self-evaluation and belief in Online fake news, we tested both full mediation and partial mediation models, comparing them. The △χ2 test showed that the partial mediation model had a better fit (△χ2 [△df = 1] = 7.616, *p* < 0.01) than the full mediation model. We further use the Bootstrap technique to figure out the mediating role of interpersonal influence. In Table 4 are shown the results of the total effect, indirect effect, and direct effect. In particular, the total effect of the self-evaluation on the belief in fake news is negatively significant (β = −0.13; confidence interval = −0.19; −0.06) and this means that as self-evaluation increases, the frequency of believing in fakes decreases. However, if interpersonal influence is inserted as a mediator between these variables, we notice that the direct effect of self-evaluation on the belief in fake news decreases (β = −0.09; confidence interval = −0.16; −0.03), showing that part of the relationship between these two variables is explained by the interpersonal influence. In line with these results, it is possible to say that interpersonal influence plays a partial mediating role in the relationship between self-evaluation and the belief in fake news (indirect effect = −0.04, CI = −0.06; −0.02) even if the effect is quite low. Nonetheless, hypothesis 3 is supported.

### 4.4. The Moderating Effect of Stages of Change

To explore the moderating effect of stages of change, invariance tests of measurement model and structural model were conducted on two different groups (in change N = 646; not in change N = 358). Table 5 demonstrates the results of measurement invariance test conducted for the two groups. The fit indices of configural model (RMSEA = 0.039, CFI = 0.995, TLI = 0.990) and metric invariance model (RMSEA = 0.035, CFI = 0.994, TLI = 0.992) indicate that both models achieve good model fit. In addition, the X^2^ difference between both models (∆X^2^ (5) = 5.291) is insignificant (*p* = 0.38) and the differences of CFI value between both models (∆CFI = 0.001) reach the suggested standards (∆CFI < 0.01) proposed by [38], indicating that the changes caused by the different groups have only a slight impact on the measurement structure and can be neglected. Consequently, the analytical results show that metric invariance is supported and thus the multigroup analysis can be conducted.

Finally, we had tested the moderating effect of stages of change creating two nested models: unconstrained model and constrained model that were compared. The results show that there is a moderating effect caused by the different stages of change (see Table 6). In addition, the results show that in the “in change” group the path coefficient between the interpersonal influence and the belief in fake news is 0.279 *** (*p* < 0.001) while it is 0.059 (*p* = n.s.) in the “not in change” group. As expected, when people are in change and want to improve the safety and the health of their eating style, the positive relationship between interpersonal influence and the belief in fake news will be stronger, determining a greater vulnerability to fake news. This evidence underlines how the different stages of change could amplify the strength of the relationship between the predisposition to social influence and the belief in food fake news. Therefore, H4 is supported.

### 4.5. Believing in Online Food Fake News and the Interest in Changing Lifestyle: The Main Socio-Demographic Differences

To assess the association between the interest in making lifestyle healthier and different socio-demographic characteristics (gender, age, and educational level) some contingency tables were created, considering the Pearson’s Chi-square to test the significance of relation. As post-hoc, standardized residuals were inspected. Since they are asymptotically normally distributed with a mean of 0 and standard deviation of 1 under the null hypothesis of independence, as a general rule of thumb cells with an absolute value of standard residuals above ±2 can be considered to significantly contribute to the general chi-square value [39]. Results show that there is a significant main effect of age [Chi-square = 30.797 (df = 2), *p* < 0.001] on the interest in making lifestyle healthier. In particular, the results showed that among those who intend to make their lifestyle healthier there is a higher percentage of young people (≤39 years) while the elderly (≥56 years) are less predisposed to change (see Table 7). On the contrary, there does not seem to be any differences for gender [Chi-square = 0.580 (df = 1), *p* = 0.446] and for educational level [Chi-square = 0.913 (df = 2), *p* = 0.634].

Finally, to evaluate the association between the frequency of believing in food fake news and the different socio-demographic characteristics (gender, age and educational level) two Welch’s ANOVA, followed by Bonferroni post-hoc comparisons, and one Student’s Test-t were carried out.

Results show that there is no significant main effect of Age [F(2, 662.717) = 2.706; *p* = 0.068], level of education [F(2, 343.890) = 2.390; *p* = 0.093], and gender [t(987.443) = 0.639; *p* = 0.523] on the frequency of believing in online food fake news.

## 5. Discussion

This research shows how social interpersonal influence mediates the relationship between individual self-evaluation and the belief in online fake news and that, with an equal predisposition to social influence, people who are planning to change their lifestyle are more likely to believe in food-related online fake news than those who do not intend to change. These results confirm our original hypothesis that individual psychological and social factors play an important role in determining why some individuals are more vulnerable to the persuasive power of online fake news, especially in the field of food consumption and nutrition. These results underline that individuals are not merely passive receivers of information, demonstrating that this phenomenon is much more complex than it has been studied so far. These evidences confirm previous studies which indicated that to better understand why people believe in fake news a further sociological or psychological inquiry is necessary [40]. In line with these reflections, some scholars [41,42] (p. 7) found that the tendency of individuals to be sceptic or to have a higher “pseudo profound bullshit receptivity” can better explain the persuasive power of fake news than the cognitive process related to the repetition of the stimulus. Confirming the fact that this is a complex phenomenon, this study has shown how the frequency of believe in online fake news in the food sector, as reported by the participants, does not depend on their socio-demographic differences such as age, educational level, and gender. These results appear to be discordant with other studies that stated that older man with less level of education have more trust in online fake news than others people [43,44]. This difference in results can be explained by the fact that this research has studied the phenomenon of fake news considering other contexts, not related to food. They also suggest that the variables that affect the belief in fake news can be different in relation to the type of news considered (political, nutritional etc.).

Moreover, it was demonstrated that the collective opinion of others influences the evaluation of the truthfulness of some news related to food [45], underling how the predisposition to social influence could do the difference in the phenomenon of fake news. Indeed our study highlights how the individual psychological self-evaluation is mediated by the susceptibility to social influence in impacting online fake news believing. The importance of these variables is also highlighted by other studies that confirmed how the online social media site have provided new and exciting tools to practice social comparisons online [46]. For example, sharing information about shocking purchases with online friends has become an habit [47], making information a function of social comparison. In addition, other works suggested that sharing news (also false) is a method of obtaining information people need for comparison and looking for social approval [48]. In line with these findings, a recent study [8] showed that the need to be approved, to increase one’s level of self-evaluation, leads to sharing news online regardless of their authenticity.

In addition, this study added an innovative dowel to the study of fake news related to food choices, understanding the role of the motivation to change in believing in this news. First of all, our results have shown that the educational level and gender do not affect the motivation to change while the age plays an important role, highlighting how younger people are more motivated to change their lifestyle than the older ones, as confirmed by previous studies [49]. Moreover, our results showed that individual psychological or social factors do not impact people’s believing in fake news in the same way for everyone, but that it depends on the individual predisposition to change. When individuals are seeking for a change in their life, they are not only more motivated to seek information, as confirmed by others studies [16,50] but also more vulnerable to social influence and fake news. In other terms the psychological readiness to change their lifestyle may open a door in people’s life, thus interrogating them about their food and dietary plans, which can result in an increased willingness to search more information. The increased interest in information seeking, indeed, can be deleterious if it leads people to believe in all (mis)information and change their food habits to improper or inadequate diets. On the contrary, it could be turned into an opportunity if it is guided: being able to diagnose people readiness to change lifestyle and food habits are an important cue to support and engage them in their correct information-seeking behaviors and improvement of healthier food habits. For instance, public health institutions and food companies could catch this opportunity to establish a dialogue with the consumers and help them in finding a proper direction. In addition, it could be an opportunity to take advantage of people’s willingness to change and to be active in their choices as a key to engage them in a fruitful relationship with the other actors of the system, thus building support and trusted network [51].

In brief, these results bring a new and strong contribution to the literature debate about the persuasiveness of online food fake news. Indeed the previous research which studied this phenomenon were based on the analysis of news feeds, Facebook posts, and tweets to figure out the spread and belief of fake news [52,53], leaving out the psychological perspectives and motives related to them. Our findings propose a first snapshot about the psychological factors that are positively and negatively associated with the persuasiveness of online food fake news. Online food fake news is becoming a great threat for public health in the agri-food sector and new knowledge about the reasons of people vulnerability is important to orient public health education initiatives. Furthermore, this study shed some light on the dark side of social media by showing how they can compromise people’s eating habits and health, especially if people are in a phase of change. It is important to mention that no study has examined such associations and variables in the past. This new knowledge about the association among food fake news, psychological factors, and the motivation to change can support dieticians and doctors in their educational initiatives. Although this research produced interesting results, it has some limitations. The frequency of beliefs in fake news is based on a self-reported item: this may be biased by memory and social desirability in the survey process. Moreover, we have not considered some variables that could affect the belief of fake news such as socio-demographic characteristics (e.g., age, level of education) and Internet usage behaviors (e.g., timing of use, information platforms used). In addition, weight and body mass index were not measured and therefore it was not possible to assess the influence of obesity on believing in nutritional fake news. Finally, our study focused on the general fake news belonging to the food field without considering whether the phenomenon of believing in fake news could change based on different types of fake news in the food sector, or if this is peculiar of certain types of foods. Further research may be conducted to better scrutinize the phenomenon of fake news by introducing other very topical variables as suggested before.

## 6. Conclusions

This research highlights how the belief in online fake news in the food sector is determined by some psychological variables and by the individual predisposition to change. Specifically, the predisposition to social influence affects the belief in online fake news if people are in a phase of dietary change since they are more open and predisposed to receive information and to listen to the advice of others. These results underline that the use of algorithms to limit the spread of online fake news can only partially solve the problem as psychological understanding of the phenomenon is an important variable within the process, both to prevent the believing and spreading of fake news in the food area, and, consequently, to engage the people toward adequate food conducts. In light of this, the study underlines the urgent need to educate consumers, especially those most exposed to fake news risk, to prevent them from unhealthy eating behaviors determined by the believing in online false news.

## Figures and Tables

**Table 1:** Demographic profiles of the sample (N = 1004).

	n	%
1. Gender		
Male	497	49.5
Female	507	50.5
2. Age		
18–25	117	11.7
26–35	149	14.9
36–45	198	19.7
46–55	218	21.7
56–65	233	23.2
66–75	88	8.8
3. Level of education		
Low education level	136	13.5
Senior high	561	55.9
College or university	307	30.6
4. Main household food purchaser		
Yes, just me	527	52.5
Yes, with others	451	44.9
No	26	2.6
5. Income level		
Until 600 €	40	4
601–900 €	54	5.4
901–1200 €	95	9.5
1201–1500 €	148	14.8
1501–1800 €	129	12.8
1801–2550 €	179	17.8
2551–3550 €	146	14.6
More than 3550 €	83	8.3
Missing	130	12.9
6. Profession		
Employed	663	66
Unemployed/retired	341	34
7. Inhabited center size		
Until 10000 inhabitants	478	47.6
10/30,000 inhabitants	140	14
30/100,000 inhabitants	149	14.8
More than 100,000	230	22.9
Missing	8	0.8
8. Geographic area		
North–West	261	26
North–East	190	18.9
Centre	199	19.8
South and Islands	354	35.3

Note: € = euro.

**Table 2:** Confirmatory factor analysis properties.

Scale	Stand. Factor Loadings	SE	*p*	CR	AVE
Core Self Evaluation scale				0.84	0.64
CSES 1 (items 9, 8, 2, 5)	0.77	0.02	***		
CSES 2 (items 4, 10, 7, 3)	0.81	0.02	***		
CSES 3 (items 6, 11, 1, 12)	0.82	0.02	***		
Interpersonal Influence				0.80	0.53
Item 1	0.93	0.02	***		
Item 2	0.87	0.02	***		
Item 3	0.46	0.03	***		
Item 4	0.53	0.02	***		

Note. *** *p* < 0.001; N = 1004; X^2^ 20.619; df = 12; *p* = n.s; CFI = 0.99; TLI = 0.99; RMSEA = 0.03 (LO90 = 0.00, HI90 = 0.05). CR = composite reliability; AVE = average variance extracted; SE = standard errors.

**Table 3:** Inter-correlations between two latent variables.

	Mean	Standard Deviation	Core Self Evaluation Scale	Interpersonal Influence
Core Self Evaluation scale	3.18	0.50	*0.80*	
Interpersonal Influence	3.80	1.23	−0.17 ***	*0.73*

Note: *** *p* < 0.001.; *N* = 1004; the square roots of AVE for discriminant validity are italicized.

**Table 4:** Standardized indirect effect of the model.

Effects of the Model			Bootstrapping	
			Percentile Bootstrapping 95% CI (Confidence Interval) of the Coefficients	
	Point Estimate	Standard Error	Lower	Upper
Total effect				
Self-evaluation→Belief in fake news	−0.13	0.03	−0.19	−0.06
Indirect effect				
Self-evaluation→interpersonal influence→Belief in fake news	−0.04	0.01	−0.06	−0.02
Direct effect				
Self-evaluation→Belief in fake news	−0.09	0.03	−0.16	−0.03

Note: Mediator: interpersonal influence; estimation of 5000 bootstrap sample.

**Table 5:** The results of measurement invariance test.

MODEL	X^2^	df	∆χ^2^_(∆df)_	∆*df*	CFI	TLI	RMSEA	ΔCFI
Group “in change” (N = 646)	13.909	12	-	-	0.999	0.998	0.016	-
Group “not in change” (N = 358)	28.075	12	-	-	0.987	0.977	0.061	-
Configural model	41.984	24	-	-	0.995	0.990	0.039	-
Metric model	47.275	29	5.291 ^ns^	5	0.994	0.992	0.035	0.001

Note: ^ns^ = not significant; RMSEA = Root Mean Square Error of Approximation; CFI = Confirmatory Fit Index; TLI=Tucker-Lewis Index; df = degree of freedom.

**Table 6:** Invariance test of the two-group structural model.

MODEL	X^2^	df	∆χ^2^_(∆df)_	∆*df*	CFI	TLI	RMSEA	ΔCFI
Unconstrained model	77.172	34	-	-	0.987	0.979	0.050	-
Constrained model	86.763	35	9.591 **	1	0.985	0.976	0.054	−0.002

Note: ** *p* < 0.01; RMSEA = Root Mean Square Error of Approximation; CFI = Confirmatory Fit Index; TLI = Tucker-Lewis Index; df = degree of freedom.

**Table 7:** Differences in the interest in making lifestyle healthier for age groups.

Variables		Cell	Age Group			Row Total
			18–39	40–55	≥56	
Interest in making lifestyle healthier	Yes	Observed	243	238	169	650
		Expected	218	224.4	207.6	
		Std res.	1.7	0.9	**−2.7**	
	No	Observed	94	109	152	355
		Expected	119	122.6	113.4	
		Std res.	**−2.3**	−1.2	**3.6**	
		CT	337	347	321	

Note: CT = column total; Std res = standard residues. Cells with an absolute value of std. res >±2 are marked in bold.

## Data Availability

The data that support the findings of this study are available from the corresponding author, upon reasonable request.

## Supplementary Materials

The following are available online at https://www.mdpi.com/1660-4601/18/6/2934/s1, Supplementary Materials S1: questionnaire A: Questionnaire filled out by the sample.
