# Supplementary material for: Online Fake News about Food: Self-Evaluation, Social Influence, and the Stages of Change Moderation
Source: Int J Environ Res Public Health. 2021 Mar 12;18(6):2934. doi: 10.3390/ijerph18062934 (PMC8001592; doi:10.3390/ijerph18062934)
Supplement: Supplementary file 1 [file ijerph-18-02934-s001.pdf]

QUESTIONNAIRE

Gender

❍ Male
❍ Female

Year of birth

Region of residence

❍ Abruzzo
❍ Basilicata
❍ Calabria
❍ Campania
❍ Emilia Romagna
❍ Friuli Venezia Giulia
❍ Lazio
❍ Liguria
❍ Lombardia
❍ Marche
❍ Molise
❍ Piemonte
❍ Puglia
❍ Sardegna
❍ Sicilia
❍ Toscana
❍ Trentino Alto Adige
❍ Umbria
❍ Valle d'Aosta
❍ Veneto

Residence municipality size

❍ Up to 10000 inhabitants
❍ 10/30.000 inhabitants
❍ 30/100.000 inhabitants
❍ More than 100.000,
❍ I don’t know

Employment

❍ Employed
❍ Not employed
❍ Retired

Level of education

❍ No educational qualifications
❍ Elementary license
❍ Lower secondary school leaving certificate
❍ High school diploma
❍ Degree

Are you the main household food purchaser?

❍ Yes, just me
❍ Yes, with others
❍ No

How interested are you in making your lifestyle healthier than it is now?

- I'm not interested in changing my lifestyle at the moment
- I plan to make changes in my lifestyle in the next 6 months
- I plan to make changes in my lifestyle this month
- I recently started making changes in my lifestyle
- I believe I already have a healthy lifestyle

In the last year have you believed in a piece of news about food read on Internet or on social networks that turned out to be a fake news (Fake News)?

❍ Never
❍ Rarely
❍ Sometimes
❍ Often
❍ Always

Indicate your degree of agreement with the following statements from 1 to 7 where:

(1 = Strongly disagree and 7=Strongly agree)

	1 Strongly disagree	2	3	4 Neither agree or disagree	5	6	7 Strongly agree
When buying products, I generally purchase those brands that I think others will approve of	☐	☐	☐	☐	☐	☐	☐
It is important that others like the products and brands I buy.	☐	☐	☐	☐	☐	☐	☐
If I have little experience with a product, I often ask my friends about the product.	☐	☐	☐	☐	☐	☐	☐
I frequently gather information from friends or family about a product before I buy.	☐	☐	☐	☐	☐	☐	☐

Indicate your degree of agreement with the following statements from 1 to 7 where:

(1 = Strongly disagree, 2 = Disagree, 3 = Neither agree or disagree, 4 = Agree, 5 = Strongly agree)

	1 Strongly disagree	2 disagree	3 Neither agree or disagree	4 Agree	5 Strongly agree
I'm sure I get the success I deserve in life	☐	☐	☐	☐	☐
Sometimes I feel sad and upset	☐	☐	☐	☐	☐
When I try, I generally succeed	☐	☐	☐	☐	☐
Sometimes when I fail I feel like I'm worth nothing	☐	☐	☐	☐	☐
I successfully complete my assignments	☐	☐	☐	☐	☐
Sometimes, I feel like I'm not in control of my job	☐	☐	☐	☐	☐
Overall, I am satisfied with myself	☐	☐	☐	☐	☐
I am full of doubts about my competence	☐	☐	☐	☐	☐

	1 Strongly disagree	2 disagree	3 Neither agree or disagree	4 Agree	5 Strongly agree
I determine what will happen in my life	☐	☐	☐	☐	☐
I feel like I am not in control of my success in my career	☐	☐	☐	☐	☐
I am able to deal with most of my problems	☐	☐	☐	☐	☐
There are times when things seem rather depressing and hopeless to me	☐	☐	☐	☐	☐

In which of these classes does your / your family's monthly net income fall?

❍ Up to 600 euro
❍ 601-900 euro
❍ 901-1200 euro
❍ 1201-1500 euro
❍ 1501-1800 euro
❍ 1801-2550 euro
❍ 2551-3550 euro
❍ More then 3550 euro
❍ I prefer not to answer
